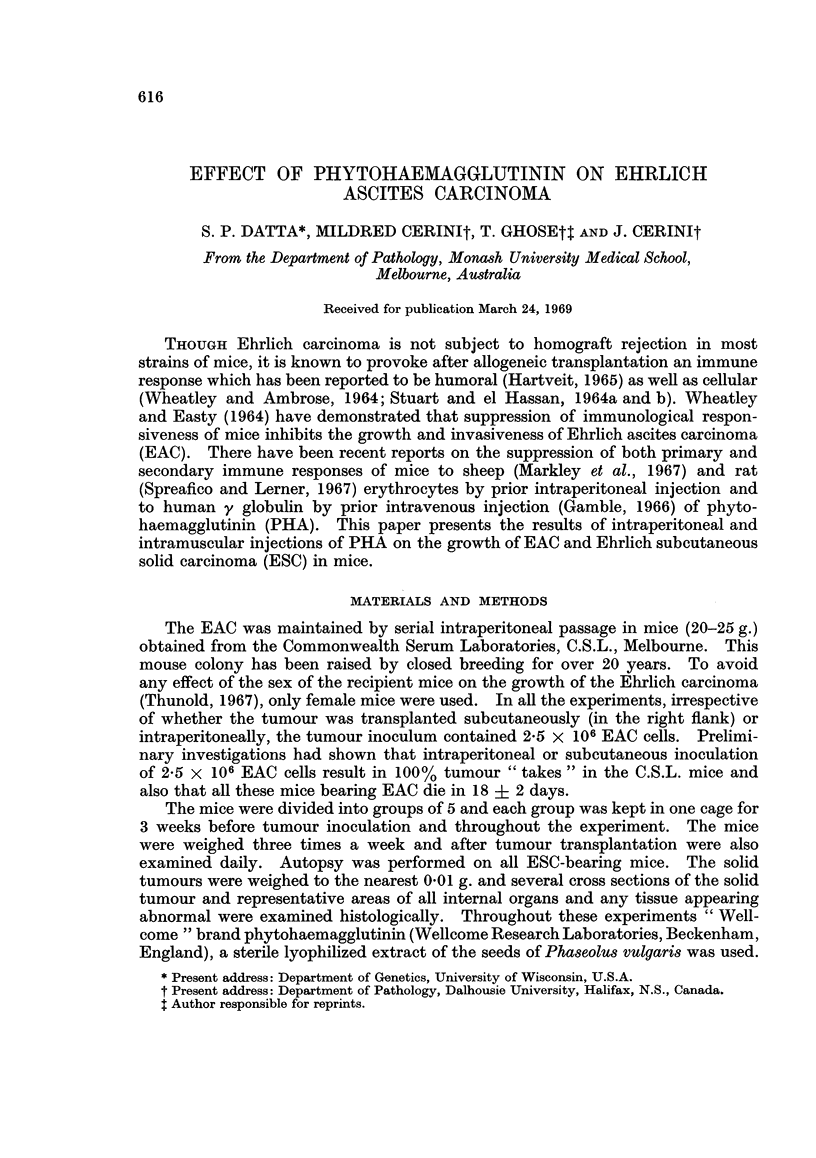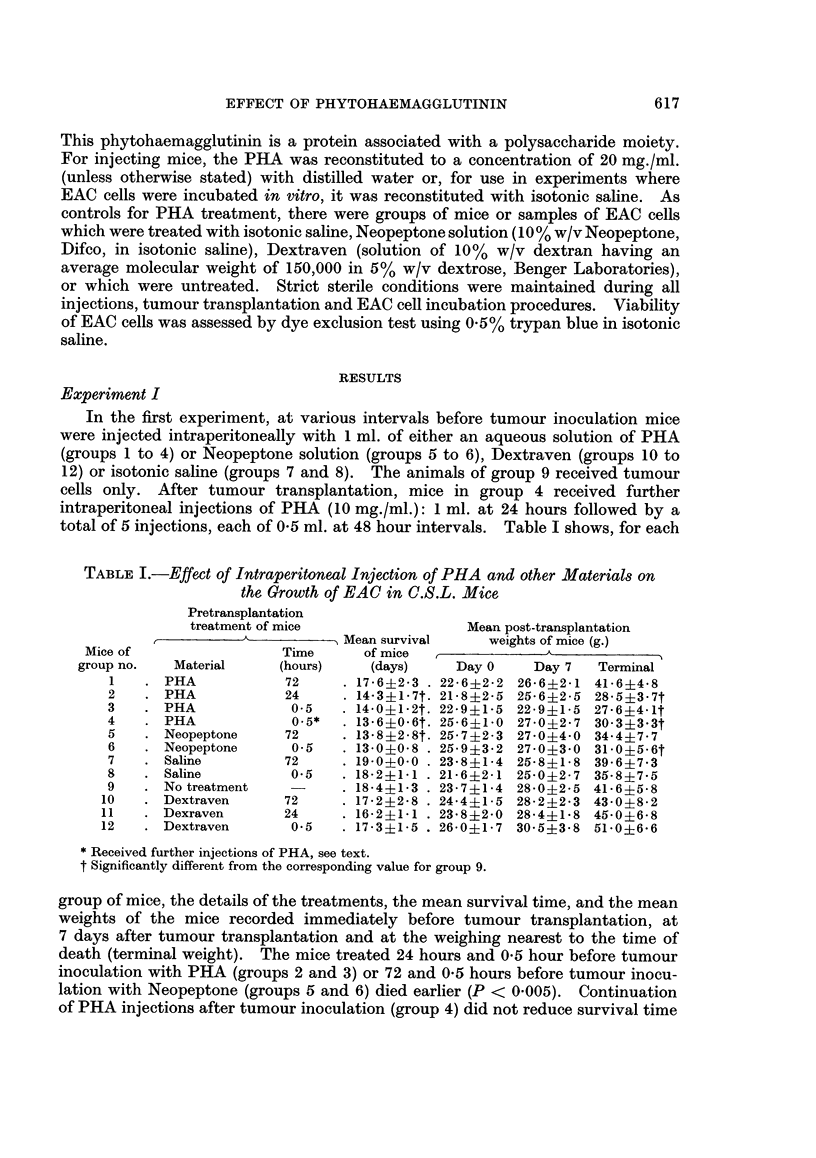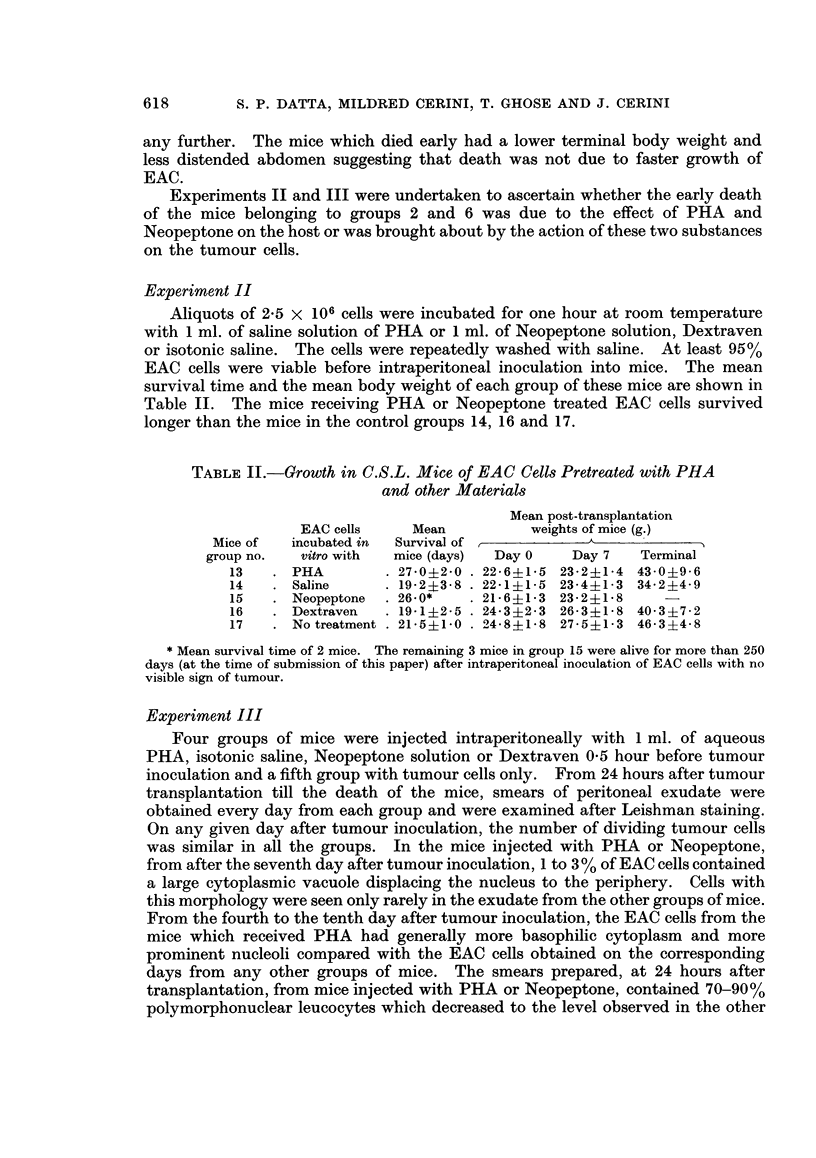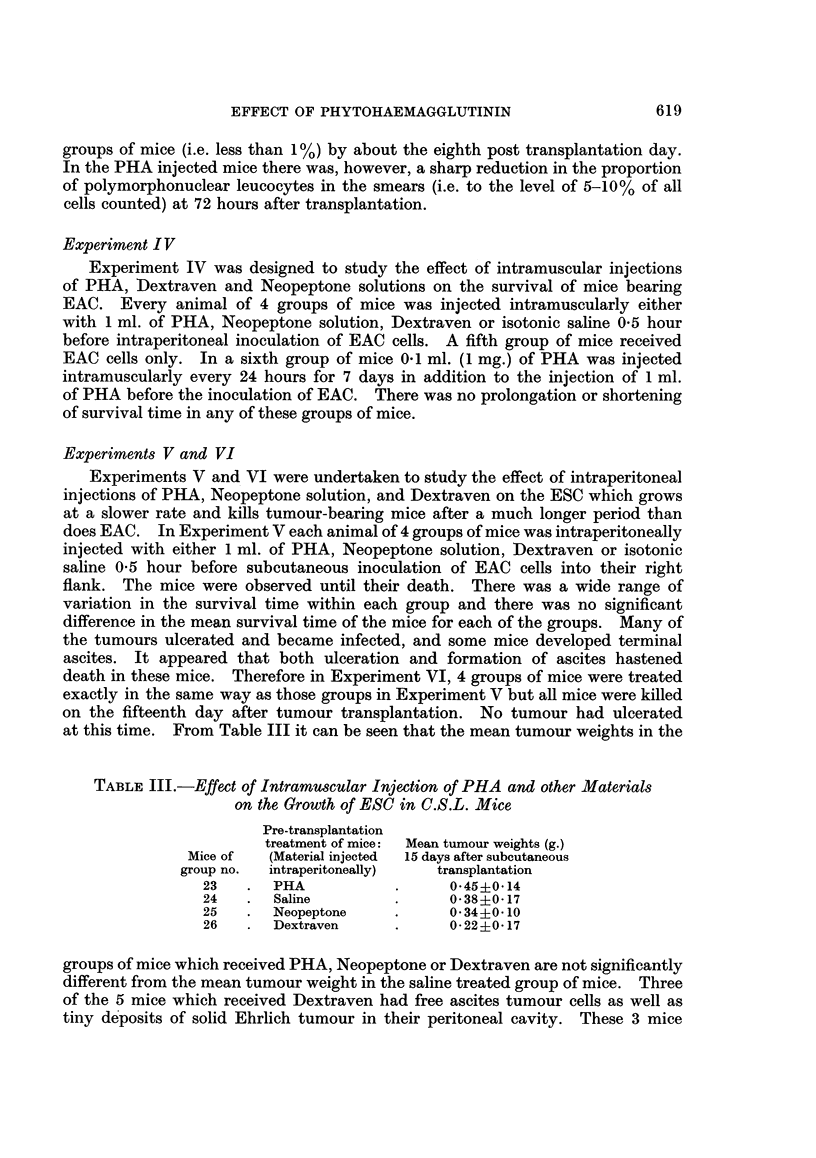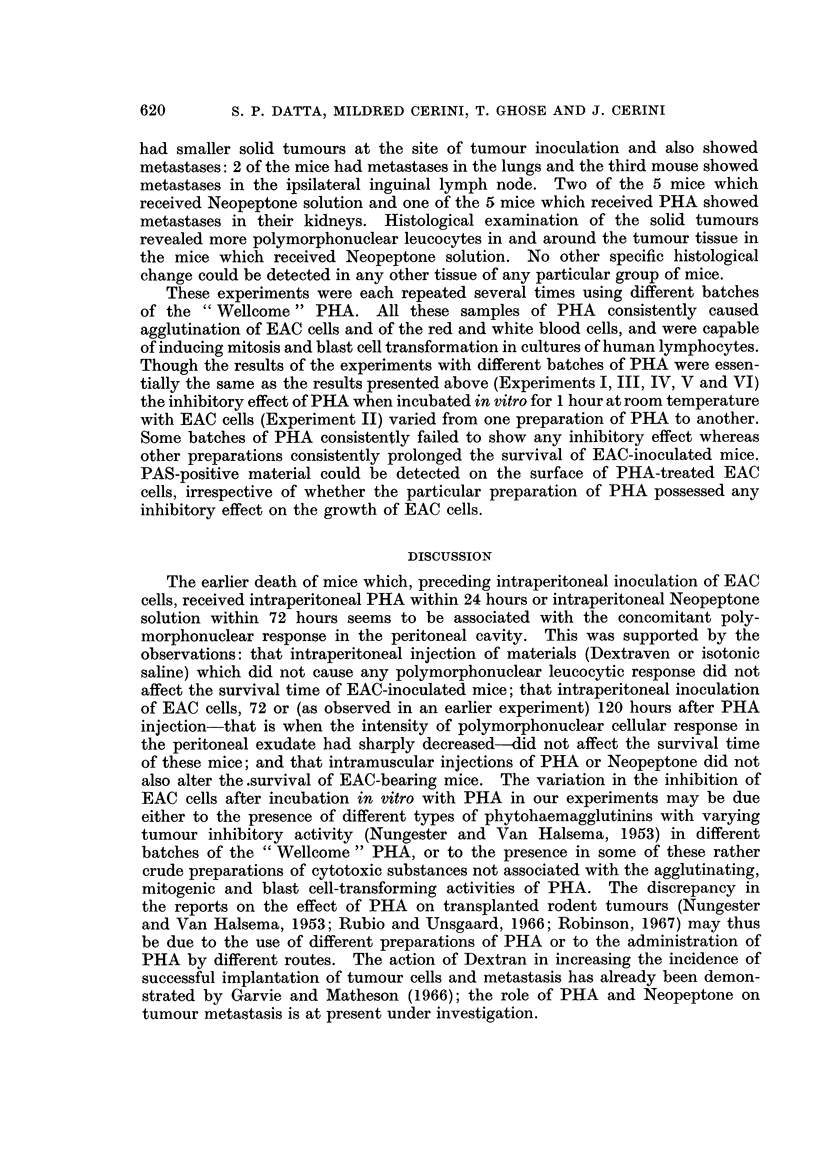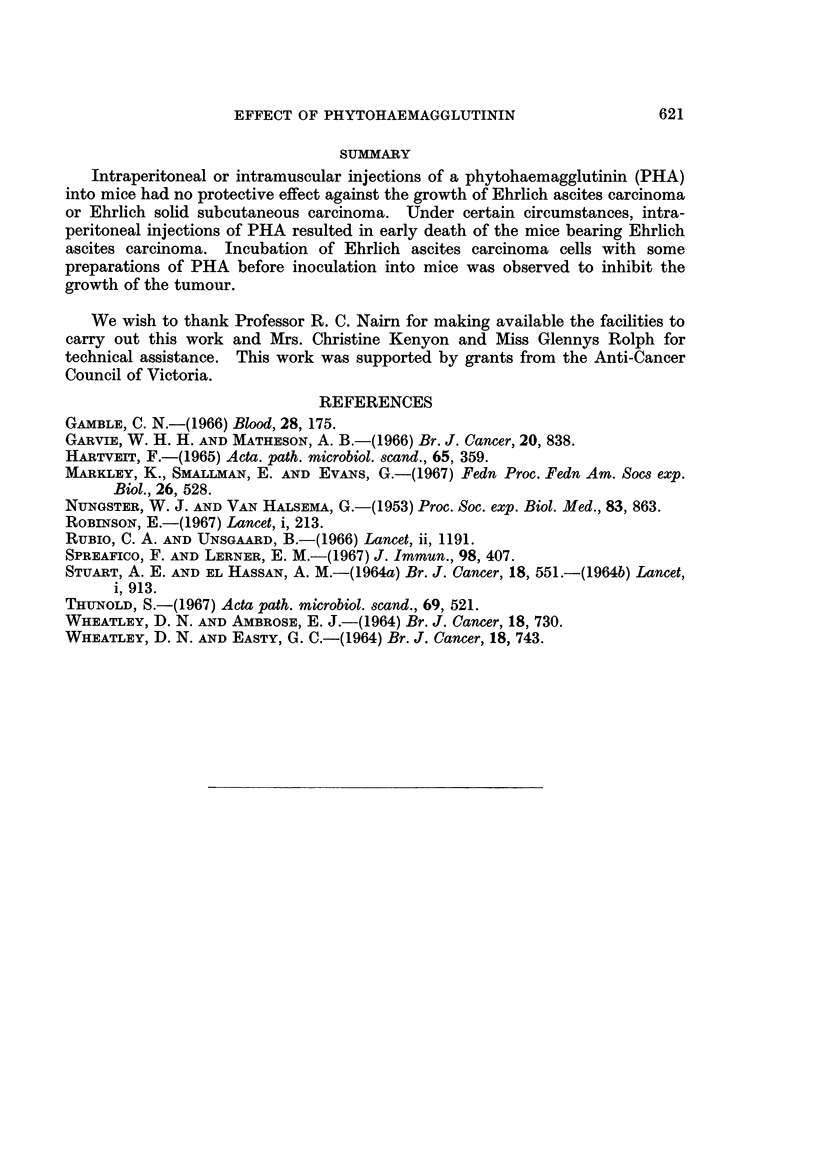# Effect of phytohaemagglutinin on Ehrlich ascites carcinoma.

**DOI:** 10.1038/bjc.1969.75

**Published:** 1969-09

**Authors:** S. P. Datta, M. Cerini, T. Ghose, J. Cerini


### EFFECT OF PHYTOHAEMAGGLUTININ ON EHRLICH ASCITES CARCINOMA

S. P. DATTA*, MILDRED CERINI†, T. GHOSE†‡ AND J. CERINI†

*From the Department of Pathology, Monash University Medical School, Melbourne, Australia*

Received for publication March 24, 1969

THOUGH Ehrlich carcinoma is not subject to homograft rejection in most strains of mice, it is known to provoke after allogeneic transplantation an immune response which has been reported to be humoral (Hartveit, 1965) as well as cellular (Wheatley and Ambrose, 1964; Stuart and el Hassan, 1964a and b). Wheatley and Easty (1964) have demonstrated that suppression of immunological responsiveness of mice inhibits the growth and invasiveness of Ehrlich ascites carcinoma (EAC). There have been recent reports on the suppression of both primary and secondary immune responses of mice to sheep (Markley *et al.*, 1967) and rat (Spreafico and Lerner, 1967) erythrocytes by prior intraperitoneal injection and to human $\gamma$ globulin by prior intravenous injection (Gamble, 1966) of phytohaemagglutinin (PHA). This paper presents the results of intraperitoneal and intramuscular injections of PHA on the growth of EAC and Ehrlich subcutaneous solid carcinoma (ESC) in mice.

### MATERIALS AND METHODS

The EAC was maintained by serial intraperitoneal passage in mice (20–25 g.) obtained from the Commonwealth Serum Laboratories, C.S.L., Melbourne. This mouse colony has been raised by closed breeding for over 20 years. To avoid any effect of the sex of the recipient mice on the growth of the Ehrlich carcinoma (Thunold, 1967), only female mice were used. In all the experiments, irrespective of whether the tumour was transplanted subcutaneously (in the right flank) or intraperitoneally, the tumour inoculum contained $2{\cdot}5 \times 10^6$ EAC cells. Preliminary investigations had shown that intraperitoneal or subcutaneous inoculation of $2{\cdot}5 \times 10^6$ EAC cells result in 100% tumour " takes " in the C.S.L. mice and also that all these mice bearing EAC die in $18 \pm 2$ days.

The mice were divided into groups of 5 and each group was kept in one cage for 3 weeks before tumour inoculation and throughout the experiment. The mice were weighed three times a week and after tumour transplantation were also examined daily. Autopsy was performed on all ESC-bearing mice. The solid tumours were weighed to the nearest 0·01 g. and several cross sections of the solid tumour and representative areas of all internal organs and any tissue appearing abnormal were examined histologically. Throughout these experiments " Wellcome " brand phytohaemagglutinin (Wellcome Research Laboratories, Beckenham, England), a sterile lyophilized extract of the seeds of *Phaseolus vulgaris* was used.

* Present address: Department of Genetics, University of Wisconsin, U.S.A.
† Present address: Department of Pathology, Dalhousie University, Halifax, N.S., Canada.
‡ Author responsible for reprints.

This phytohaemagglutinin is a protein associated with a polysaccharide moiety. For injecting mice, the PHA was reconstituted to a concentration of 20 mg./ml. (unless otherwise stated) with distilled water or, for use in experiments where EAC cells were incubated *in vitro*, it was reconstituted with isotonic saline. As controls for PHA treatment, there were groups of mice or samples of EAC cells which were treated with isotonic saline, Neopeptone solution (10% w/v Neopeptone, Difco, in isotonic saline), Dextraven (solution of 10% w/v dextran having an average molecular weight of 150,000 in 5% w/v dextrose, Benger Laboratories), or which were untreated. Strict sterile conditions were maintained during all injections, tumour transplantation and EAC cell incubation procedures. Viability of EAC cells was assessed by dye exclusion test using 0·5% trypan blue in isotonic saline.

### RESULTS

### *Experiment I*

In the first experiment, at various intervals before tumour inoculation mice were injected intraperitoneally with 1 ml. of either an aqueous solution of PHA (groups 1 to 4) or Neopeptone solution (groups 5 to 6), Dextraven (groups 10 to 12) or isotonic saline (groups 7 and 8). The animals of group 9 received tumour cells only. After tumour transplantation, mice in group 4 received further intraperitoneal injections of PHA (10 mg./ml.): 1 ml. at 24 hours followed by a total of 5 injections, each of 0·5 ml. at 48 hour intervals. Table I shows, for each

TABLE I.—*Effect of Intraperitoneal Injection of PHA and other Materials on the Growth of EAC in C.S.L. Mice*

| Mice of group no. | Pretransplantation treatment of mice: Material | Time (hours) | Mean survival of mice (days) | Mean post-transplantation weights of mice (g.): Day 0 | Day 7 | Terminal |
|---|---|---|---|---|---|---|
| 1 | PHA | 72 | 17·6±2·3 | 22·6±2·2 | 26·6±2·1 | 41·6±4·8 |
| 2 | PHA | 24 | 14·3±1·7† | 21·8±2·5 | 25·6±2·5 | 28·5±3·7† |
| 3 | PHA | 0·5 | 14·0±1·2† | 22·9±1·5 | 22·9±1·5 | 27·6±4·1† |
| 4 | PHA | 0·5* | 13·6±0·6† | 25·6±1·0 | 27·0±2·7 | 30·3±3·3† |
| 5 | Neopeptone | 72 | 13·8±2·8† | 25·7±2·3 | 27·0±4·0 | 34·4±7·7 |
| 6 | Neopeptone | 0·5 | 13·0±0·8 | 25·9±3·2 | 27·0±3·0 | 31·0±5·6† |
| 7 | Saline | 72 | 19·0±0·0 | 23·8±1·4 | 25·8±1·8 | 39·6±7·3 |
| 8 | Saline | 0·5 | 18·2±1·1 | 21·6±2·1 | 25·0±2·7 | 35·8±7·5 |
| 9 | No treatment | — | 18·4±1·3 | 23·7±1·4 | 28·0±2·5 | 41·6±5·8 |
| 10 | Dextraven | 72 | 17·2±2·8 | 24·4±1·5 | 28·2±2·3 | 43·0±8·2 |
| 11 | Dexraven | 24 | 16·2±1·1 | 23·8±2·0 | 28·4±1·8 | 45·0±6·8 |
| 12 | Dextraven | 0·5 | 17·3±1·5 | 26·0±1·7 | 30·5±3·8 | 51·0±6·6 |

* Received further injections of PHA, see text.
† Significantly different from the corresponding value for group 9.

group of mice, the details of the treatments, the mean survival time, and the mean weights of the mice recorded immediately before tumour transplantation, at 7 days after tumour transplantation and at the weighing nearest to the time of death (terminal weight). The mice treated 24 hours and 0·5 hour before tumour inoculation with PHA (groups 2 and 3) or 72 and 0·5 hours before tumour inoculation with Neopeptone (groups 5 and 6) died earlier ($P < 0{\cdot}005$). Continuation of PHA injections after tumour inoculation (group 4) did not reduce survival time any further. The mice which died early had a lower terminal body weight and less distended abdomen suggesting that death was not due to faster growth of EAC.

Experiments II and III were undertaken to ascertain whether the early death of the mice belonging to groups 2 and 6 was due to the effect of PHA and Neopeptone on the host or was brought about by the action of these two substances on the tumour cells.

*Experiment II*

Aliquots of $2 \cdot 5 \times 10^6$ cells were incubated for one hour at room temperature with 1 ml. of saline solution of PHA or 1 ml. of Neopeptone solution, Dextraven or isotonic saline. The cells were repeatedly washed with saline. At least 95% EAC cells were viable before intraperitoneal inoculation into mice. The mean survival time and the mean body weight of each group of these mice are shown in Table II. The mice receiving PHA or Neopeptone treated EAC cells survived longer than the mice in the control groups 14, 16 and 17.

TABLE II.—*Growth in C.S.L. Mice of EAC Cells Pretreated with PHA and other Materials*

| Mice of group no. | EAC cells incubated *in vitro* with | Mean Survival of mice (days) | Mean post-transplantation weights of mice (g.) | | |
|---|---|---|---|---|---|
| | | | Day 0 | Day 7 | Terminal |
| 13 | PHA | $27 \cdot 0 \pm 2 \cdot 0$ | $22 \cdot 6 \pm 1 \cdot 5$ | $23 \cdot 2 \pm 1 \cdot 4$ | $43 \cdot 0 \pm 9 \cdot 6$ |
| 14 | Saline | $19 \cdot 2 \pm 3 \cdot 8$ | $22 \cdot 1 \pm 1 \cdot 5$ | $23 \cdot 4 \pm 1 \cdot 3$ | $34 \cdot 2 \pm 4 \cdot 9$ |
| 15 | Neopeptone | 26·0* | $21 \cdot 6 \pm 1 \cdot 3$ | $23 \cdot 2 \pm 1 \cdot 8$ | — |
| 16 | Dextraven | $19 \cdot 1 \pm 2 \cdot 5$ | $24 \cdot 3 \pm 2 \cdot 3$ | $26 \cdot 3 \pm 1 \cdot 8$ | $40 \cdot 3 \pm 7 \cdot 2$ |
| 17 | No treatment | $21 \cdot 5 \pm 1 \cdot 0$ | $24 \cdot 8 \pm 1 \cdot 8$ | $27 \cdot 5 \pm 1 \cdot 3$ | $46 \cdot 3 \pm 4 \cdot 8$ |

* Mean survival time of 2 mice. The remaining 3 mice in group 15 were alive for more than 250 days (at the time of submission of this paper) after intraperitoneal inoculation of EAC cells with no visible sign of tumour.

*Experiment III*

Four groups of mice were injected intraperitoneally with 1 ml. of aqueous PHA, isotonic saline, Neopeptone solution or Dextraven 0·5 hour before tumour inoculation and a fifth group with tumour cells only. From 24 hours after tumour transplantation till the death of the mice, smears of peritoneal exudate were obtained every day from each group and were examined after Leishman staining. On any given day after tumour inoculation, the number of dividing tumour cells was similar in all the groups. In the mice injected with PHA or Neopeptone, from after the seventh day after tumour inoculation, 1 to 3% of EAC cells contained a large cytoplasmic vacuole displacing the nucleus to the periphery. Cells with this morphology were seen only rarely in the exudate from the other groups of mice. From the fourth to the tenth day after tumour inoculation, the EAC cells from the mice which received PHA had generally more basophilic cytoplasm and more prominent nucleoli compared with the EAC cells obtained on the corresponding days from any other groups of mice. The smears prepared, at 24 hours after transplantation, from mice injected with PHA or Neopeptone, contained 70–90% polymorphonuclear leucocytes which decreased to the level observed in the other groups of mice (i.e. less than 1%) by about the eighth post transplantation day. In the PHA injected mice there was, however, a sharp reduction in the proportion of polymorphonuclear leucocytes in the smears (i.e. to the level of 5–10% of all cells counted) at 72 hours after transplantation.

*Experiment IV*

Experiment IV was designed to study the effect of intramuscular injections of PHA, Dextraven and Neopeptone solutions on the survival of mice bearing EAC. Every animal of 4 groups of mice was injected intramuscularly either with 1 ml. of PHA, Neopeptone solution, Dextraven or isotonic saline 0·5 hour before intraperitoneal inoculation of EAC cells. A fifth group of mice received EAC cells only. In a sixth group of mice 0·1 ml. (1 mg.) of PHA was injected intramuscularly every 24 hours for 7 days in addition to the injection of 1 ml. of PHA before the inoculation of EAC. There was no prolongation or shortening of survival time in any of these groups of mice.

*Experiments V and VI*

Experiments V and VI were undertaken to study the effect of intraperitoneal injections of PHA, Neopeptone solution, and Dextraven on the ESC which grows at a slower rate and kills tumour-bearing mice after a much longer period than does EAC. In Experiment V each animal of 4 groups of mice was intraperitoneally injected with either 1 ml. of PHA, Neopeptone solution, Dextraven or isotonic saline 0·5 hour before subcutaneous inoculation of EAC cells into their right flank. The mice were observed until their death. There was a wide range of variation in the survival time within each group and there was no significant difference in the mean survival time of the mice for each of the groups. Many of the tumours ulcerated and became infected, and some mice developed terminal ascites. It appeared that both ulceration and formation of ascites hastened death in these mice. Therefore in Experiment VI, 4 groups of mice were treated exactly in the same way as those groups in Experiment V but all mice were killed on the fifteenth day after tumour transplantation. No tumour had ulcerated at this time. From Table III it can be seen that the mean tumour weights in the

TABLE III.—*Effect of Intramuscular Injection of PHA and other Materials on the Growth of ESC in C.S.L. Mice*

| Mice of group no. | Pre-transplantation treatment of mice: (Material injected intraperitoneally) | Mean tumour weights (g.) 15 days after subcutaneous transplantation |
|---|---|---|
| 23 | PHA | $0 \cdot 45 \pm 0 \cdot 14$ |
| 24 | Saline | $0 \cdot 38 \pm 0 \cdot 17$ |
| 25 | Neopeptone | $0 \cdot 34 \pm 0 \cdot 10$ |
| 26 | Dextraven | $0 \cdot 22 \pm 0 \cdot 17$ |

groups of mice which received PHA, Neopeptone or Dextraven are not significantly different from the mean tumour weight in the saline treated group of mice. Three of the 5 mice which received Dextraven had free ascites tumour cells as well as tiny deposits of solid Ehrlich tumour in their peritoneal cavity. These 3 mice had smaller solid tumours at the site of tumour inoculation and also showed metastases: 2 of the mice had metastases in the lungs and the third mouse showed metastases in the ipsilateral inguinal lymph node. Two of the 5 mice which received Neopeptone solution and one of the 5 mice which received PHA showed metastases in their kidneys. Histological examination of the solid tumours revealed more polymorphonuclear leucocytes in and around the tumour tissue in the mice which received Neopeptone solution. No other specific histological change could be detected in any other tissue of any particular group of mice.

These experiments were each repeated several times using different batches of the " Wellcome " PHA. All these samples of PHA consistently caused agglutination of EAC cells and of the red and white blood cells, and were capable of inducing mitosis and blast cell transformation in cultures of human lymphocytes. Though the results of the experiments with different batches of PHA were essentially the same as the results presented above (Experiments I, III, IV, V and VI) the inhibitory effect of PHA when incubated *in vitro* for 1 hour at room temperature with EAC cells (Experiment II) varied from one preparation of PHA to another. Some batches of PHA consistently failed to show any inhibitory effect whereas other preparations consistently prolonged the survival of EAC-inoculated mice. PAS-positive material could be detected on the surface of PHA-treated EAC cells, irrespective of whether the particular preparation of PHA possessed any inhibitory effect on the growth of EAC cells.

### DISCUSSION

The earlier death of mice which, preceding intraperitoneal inoculation of EAC cells, received intraperitoneal PHA within 24 hours or intraperitoneal Neopeptone solution within 72 hours seems to be associated with the concomitant polymorphonuclear response in the peritoneal cavity. This was supported by the observations: that intraperitoneal injection of materials (Dextraven or isotonic saline) which did not cause any polymorphonuclear leucocytic response did not affect the survival time of EAC-inoculated mice; that intraperitoneal inoculation of EAC cells, 72 or (as observed in an earlier experiment) 120 hours after PHA injection—that is when the intensity of polymorphonuclear cellular response in the peritoneal exudate had sharply decreased—did not affect the survival time of these mice; and that intramuscular injections of PHA or Neopeptone did not also alter the survival of EAC-bearing mice. The variation in the inhibition of EAC cells after incubation *in vitro* with PHA in our experiments may be due either to the presence of different types of phytohaemagglutinins with varying tumour inhibitory activity (Nungester and Van Halsema, 1953) in different batches of the " Wellcome " PHA, or to the presence in some of these rather crude preparations of cytotoxic substances not associated with the agglutinating, mitogenic and blast cell-transforming activities of PHA. The discrepancy in the reports on the effect of PHA on transplanted rodent tumours (Nungester and Van Halsema, 1953; Rubio and Unsgaard, 1966; Robinson, 1967) may thus be due to the use of different preparations of PHA or to the administration of PHA by different routes. The action of Dextran in increasing the incidence of successful implantation of tumour cells and metastasis has already been demonstrated by Garvie and Matheson (1966); the role of PHA and Neopeptone on tumour metastasis is at present under investigation.

### SUMMARY

Intraperitoneal or intramuscular injections of a phytohaemagglutinin (PHA) into mice had no protective effect against the growth of Ehrlich ascites carcinoma or Ehrlich solid subcutaneous carcinoma. Under certain circumstances, intraperitoneal injections of PHA resulted in early death of the mice bearing Ehrlich ascites carcinoma. Incubation of Ehrlich ascites carcinoma cells with some preparations of PHA before inoculation into mice was observed to inhibit the growth of the tumour.

We wish to thank Professor R. C. Nairn for making available the facilities to carry out this work and Mrs. Christine Kenyon and Miss Glennys Rolph for technical assistance. This work was supported by grants from the Anti-Cancer Council of Victoria.

### REFERENCES

GAMBLE, C. N.—(1966) *Blood*, **28**, 175.
GARVIE, W. H. H. AND MATHESON, A. B.—(1966) *Br. J. Cancer*, **20**, 838.
HARTVEIT, F.—(1965) *Acta. path. microbiol. scand.*, **65**, 359.
MARKLEY, K., SMALLMAN, E. AND EVANS, G.—(1967) *Fedn Proc. Fedn Am. Socs exp. Biol.*, **26**, 528.
NUNGSTER, W. J. AND VAN HALSEMA, G.—(1953) *Proc. Soc. exp. Biol. Med.*, **83**, 863.
ROBINSON, E.—(1967) *Lancet*, i, 213.
RUBIO, C. A. AND UNSGAARD, B.—(1966) *Lancet*, ii, 1191.
SPREAFICO, F. AND LERNER, E. M.—(1967) *J. Immun.*, **98**, 407.
STUART, A. E. AND EL HASSAN, A. M.—(1964*a*) *Br. J. Cancer*, **18**, 551.—(1964*b*) *Lancet*, i, 913.
THUNOLD, S.—(1967) *Acta path. microbiol. scand.*, **69**, 521.
WHEATLEY, D. N. AND AMBROSE, E. J.—(1964) *Br. J. Cancer*, **18**, 730.
WHEATLEY, D. N. AND EASTY, G. C.—(1964) *Br. J. Cancer*, **18**, 743.